# Swine methicillin-resistant *Staphylococcus aureus* carrying toxic-shock syndrome toxin gene in Hong Kong, China

**DOI:** 10.1080/22221751.2020.1785335

**Published:** 2020-07-07

**Authors:** Dulmini Nanayakkara Sapugahawatte, Carmen Li, Yun Kit Yeoh, Priyanga Dharmaratne, Chendi Zhu, Margaret Ip

**Affiliations:** a Department of Microbiology, Faculty of Medicine, The Chinese University of Hong Kong, Prince of Wales Hospital, Hong Kong (SAR), People’s Republic of China; b School of Biomedical Sciences, The Chinese University of Hong Kong, Hong Kong (SAR), People’s Republic of China

**Keywords:** Antimicrobial resistance, staphylococci, zoonoses, swine, Asia, MRSA, Hong Kong, toxic shock syndrome (*tsst-1*)

## Abstract

We report a SCC*mec* II, ST39 methicillin-resistant *Staphylococcus aureus* isolate from pigs that harboured toxic-shock syndrome toxin gene (*tsst-*1). The gene was located in a rare pathogenicity island SaPI68111, which also carried enterotoxin genes that can cause fatal infections. Pigs may potentially serve as a reservoir for MRSA dissemination.

Livestock associated Methicillin-resistant *Staphylococcus aureus* (LA-MRSA) has been observed in human host as carriage strains and can cause infections [1]. LA-MRSA has distinct geographical lineages, namely sequence type (ST) 9 in Asia and ST398 in Europe [2]. We report for the first time 2 ST39 MRSA strains harbouring toxic shock syndrome gene (*tsst-1*) from pig tongues in Hong Kong.

Twenty-one MRSA isolates were identified from 191 pig tongues (10.9%) in our food surveillance for antimicrobial resistance from raw meat products purchased from 18 markets of Hong Kong between April 2018 and May 2019. Measures were taken to minimize cross-contamination between specimens. Briefly, two grams of pig tongue was dissected, macerated and inoculated in Contrast MRSA Broth for overnight incubation at 37°C prior to seeding onto MRSA CHROMagar (BioMérieux, France). Bacterial species were identified by matrix-assisted laser desorption/ionization time of flight mass spectrometer (MALDI-TOF-MS) (Bruker-Daltonics, Germany). All the confirmed *Staphylococcus aureus* strains were checked for the presence of *mecA* gene by PCR and confirmed as MRSA [1]. All the pig MRSA strains were subjected to Pulse Field Gel Electrophoresis (PFGE) (https://www.cdc.gov/hai/pdfs/labsettings/unified_pfge_protocol.pdf) after digestion with *Sma*I enzyme. Control strain *S. aureus* ATCC25923 was also included. Multilocus sequence typing (MLST) was performed according to pubMLST protocol (www.pubmlst.org). All the ST39, ST398 and about 50% of all ST9 strains were selected for whole genome sequencing (WGS). Genomic DNA was extracted using Wizard Genomic DNA kit (Promega, USA) followed by library preparation and sequencing with Rapid DNA library prep kit (iGenomx, USA) and Illumina NextSeq 500 platform (Illumina, USA) respectively according to manufacturers’ protocol to give 50x average coverage. Quality control, *de novo* assembly of the sequence reads were performed as previously described [3]. The presence of acquired antibiotic resistance genes, putative host plasmids and SCCmec prediction were determined by ResFinder 3.1, PlasmidFinder 2.1 and SCCmecFinder respectively from Center for Genomic Epidemiology homepage (https://cge.cbs.dtu.dk/services/). Gene alignment and annotation was conducted by Geneious (v11.0) (Biomatters Ltd, New Zealand). Availability of *tsst-1* gene was confirmed with PCR assay [4].

All the 21 strains in this study carried *mecA* gene. Of the 21 strains, 16 strains belonged to ST9, strain number m87 was ST398, m97 was ST5714 and 2 strains (m11 and m95) were ST39. ST typing was not done (ND) in one of the strains that fell within the PFGE cluster belonging to ST9. Moreover, PFGE analysis at 80% confidence level revealed 3 clusters of MRSA that differentiated ST9 from other STs (Figure 1). ST39 and ST398 were the remaining 2 clusters where ST398 is untypable by *Sma*I enzyme in PFGE [5] as the chromosome is methylated and resist digestion at the *Sma*I restriction site. ST39 is a double-locus variant of ST30, the founder sequence of the clonal complex (CC) 30. Both ST39 isolates belonged to SCCmec type II, *spa* type t007 and were phylogenetically distant from ST9 and ST398. All strains carried genes encoding capsules (*cap*) and iron-regulated surface determinant (*Isd*) but were negative for Panton-Valentine leucocidin (PVL) and exfoliative toxins. Genes encoding adhesins and hemolysins were common. Notably, genes conferring enterotoxins, toxic-shock syndrome toxin, staphylokinase and human immune evasion clusters were exclusively present in ST39 MRSA isolates (Figure S1). More than half of the isolates carried resistance genes for β-lactams (*blaZ*), fosfomycin (*fosD*), lincosamide (*InuB*), chloramphenicol (*fexA*), sulfonamide (*dfrG*), tetracyclines (*tetL*) and macrolides (*ermC* in ST9 *and ermA* in ST39). Additional resistance genes were found in ST39 isolates against streptomycin (*spc*), and aminoglycoside (*aadD*) (Figure S1). ST39 strains carried more plasmids than our ST9 lineage, where plasmids pSJH101, pMW2 and pLW043 were exclusively present in ST39. Of note, m11 carried an additional plasmid SAP019A plasmid encoding a truncated transposase (Figure S1). *Tsst-1* gene in m11 and m95 was located in a rare 16kbp pathogenicity island SaPI68111 which also harboured two enterotoxin genes *sec*3 and *sel* (unpublished). SaPI68111 was once identified from a methicillin-sensitive *S. aureus* (MSSA) strain that led to fatal pneumonia in an immunocompetent child [6]. To date, SaPI68111 has not been reported in ST39 lineage or in livestock.

**Figure 1.**
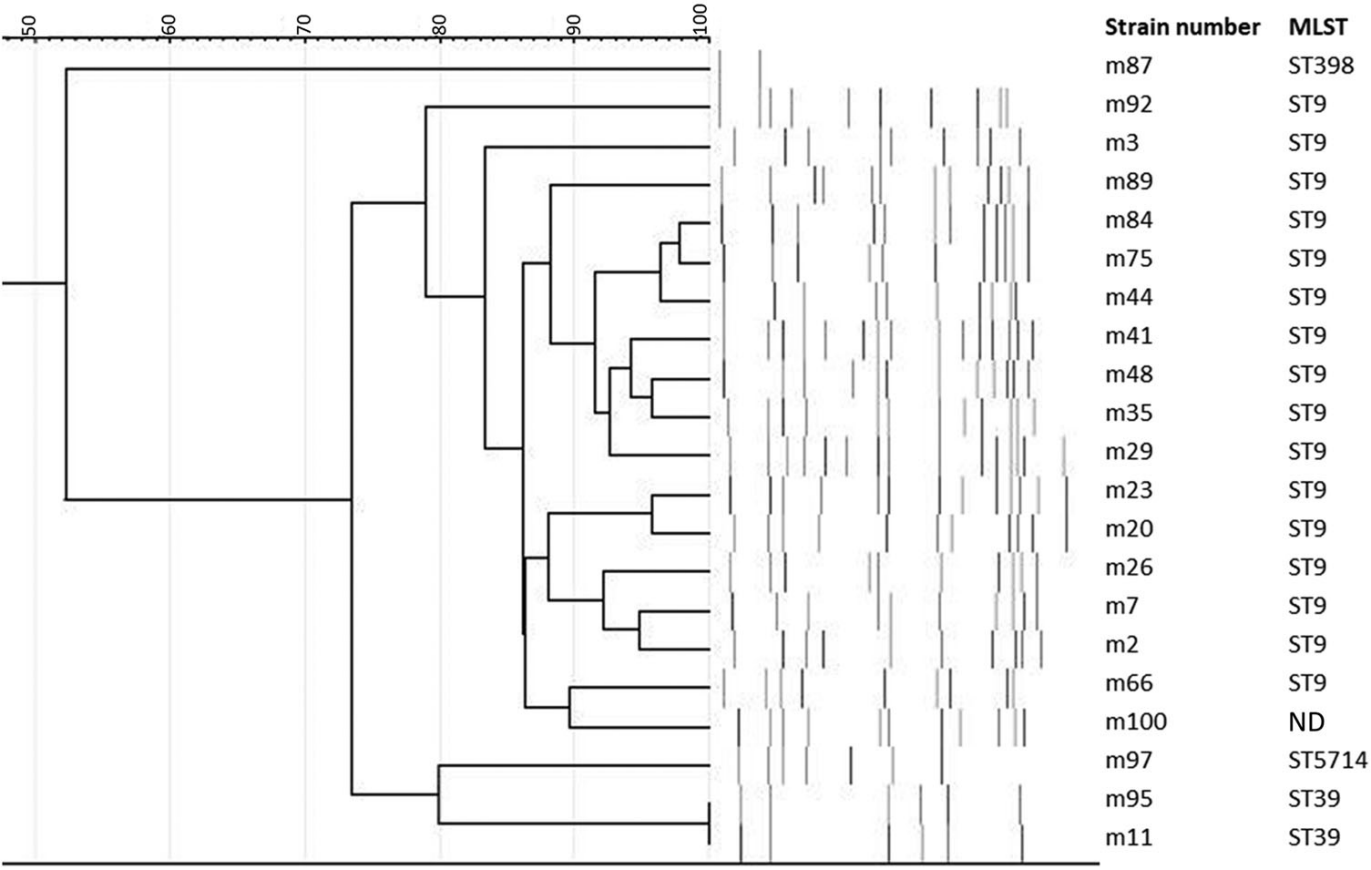
Dendrogram and PFGE profile observed among 21 pig MRSA strains. The similarities between the fingerprints were calculated using the Dice coefficient (optimization, 1%; position tolerance, 0.1% to 1%), and the fingerprints were grouped according to their similarities by use of the UPGMA algorithm. ND; Not done.

ST39 MRSA appeared to interact with both humans and animals. It was observed in community-acquired infections in humans in Iran [7] and was also reported in food animals, such as seafood in India [8] and pigs [9]. We screened through our 132 bacteremic MRSA isolates from blood culture for the same period as this study to see whether ST39 *tsst-1* was common. Only 1 isolate carried *tsst-1* gene and was ST672 (unpublished), thus it was not further investigated. ST39 MSSA carrying *tsst-1* gene was reported in Germany causing human infections, but this clone belonged to *spa* type t339 [10]. Two years later, the European Food Safety Authority reported ST39-t007 MRSA colonized breeding pigs but were *tsst-1* negative [9]. Our findings from retail pigs suggest that *tsst-1* positive ST39 MRSA has emerged in pigs in Asia besides that seen in ST9.

In conclusion, ST39 MRSA harbouring *tsst-1* have been identified in pigs. This superantigen co-exists with enterotoxin genes which are associated with staphylococcal food poisoning. The presence of human immune evasion cluster in this lineage indicates the possibility to cause human infection and a potential emergence to the human and pig population.

## Supplementary Material

Supplementary_information.docx
