# Supplementary material for: Swine methicillin-resistant Staphylococcus aureus carrying toxic-shock syndrome toxin gene in Hong Kong, China
Source: Emerg Microbes Infect. 2020 Jul 7;9(1):1534–6. doi: 10.1080/22221751.2020.1785335 (PMC7473289; doi:10.1080/22221751.2020.1785335)
Supplement: Supplementary_information.docx [file TEMI_A_1785335_SM7631.docx]

Supplementary information for,

Swine Methicillin-resistant *Staphylococcus aureus* carrying toxic-shock syndrome toxin gene in Hong Kong, China.

Dulmini Nanayakkara Sapugahawatte^a^, Carmen Li^a^, Yun Kit Yeoh^a^, Priyanga Dharmaratne^b^, Margaret Ip^a^*

^a^Department of Microbiology, Faculty of Medicine, The Chinese University of Hong Kong, Prince of Wales Hospital, Sha Tin, Hong Kong (SAR), China.

^b^School of Biomedical Sciences, The Chinese University of Hong Kong, Sha Tin, Hong Kong (SAR), China.


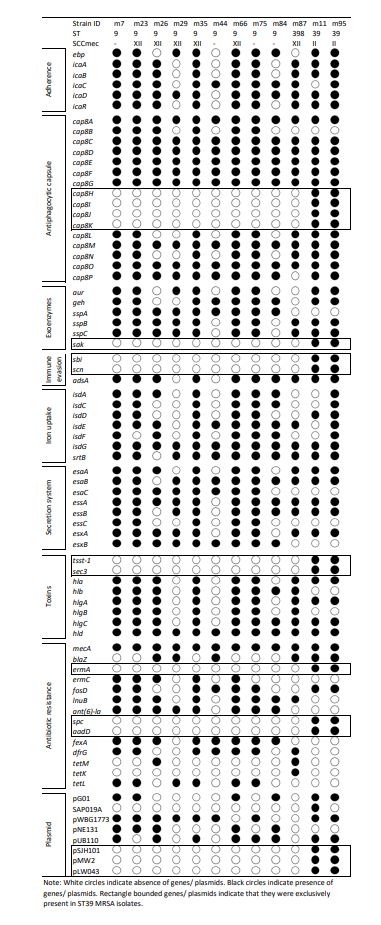


Figure S1. Molecular characteristics of 12 representative pig MRSA strains. The presence and absence of genes or plasmids of a strain are indicated as white and black circles respectively. Genes or plasmids that were exclusively present in ST39 MRSA isolates were bounded with a rectangle.
